# The Effect of Equine Assisted Learning on Improving Stress, Health, and Coping among Quarantine Control Workers in South Korea

**DOI:** 10.3390/healthcare10081564

**Published:** 2022-08-18

**Authors:** Taewoon Jung, Hyoungjin Park, Jeong-Yi Kwon, Sunju Sohn

**Affiliations:** 1 Department of Physical Education, Yongin University, Yongin 17092, Korea; 2 Department of Physical Education, Humanities & Arts, Korea Science Academy of KAIST, Busan 47162, Korea; 3 Department of Physical & Rehabilitation Medicine, Sungkyunkwan University School of Medicine, Suwon 16419, Korea; 4 Department of Social Welfare, Cheongju University, Cheongju 28503, Korea

**Keywords:** equine assisted learning, stress reduction, coping, well-being, disaster control workers

## Abstract

Foot-and-Mouth Disease (FMD) and Avian Influenza (AI) frequently occur in South Korea, resulting in high levels of occupational stress among quarantine workers forced to partake in massive livestock killings. This study explored the usefulness of Equine Assisted Learning (EAL) in improving these workers’ psychological and emotional functioning. A total of 51 FMD/AI control workers participated in 16 sessions of an EAL program facilitated by therapeutic riding professionals and trained horses. Results showed significant changes in their stress level, coping style, and overall quality of life-related to health, most notably increased vitality, enhanced emotional and social functioning, greater problem-solving, and less social avoidance after EAL participation. The usefulness of equine-assisted activities and the association between more significant stress coping ability and improved functioning in various areas of life are consistent with previous research findings. Implications for EAL application are discussed.

## 1. Introduction

Little is known regarding the experiences of FMD/AI control workers; however, based on previous research findings on stress and trauma related to disaster work, we may assume the stress levels among these professionals. Disaster relief work is associated with high-stress levels, possible lingering mental health problems, burnout, and quick turnover rates [1]. When people constantly react to stressful situations without making emotional and/or psychological adjustments to counter the adverse effects, they are likely to feel stressed, endangering their health and well-being. Control workers are similar in that their tasks involve killing activities that are known to be highly stressful and may lead to trauma in some individuals. If left untreated, this can create various unstable emotional and psychological states, including stress, anxiety, depression, and post-traumatic stress disorder (PTSD) [2]. Especially for FMD/AI workers, there are discomforting symptoms that hinder job performance, such as personal fear or guilt before, during, and after the massive culling [3] and ambivalence and moral conflict between one’s values and what needs to be done as part of the job [4]. Hence, it is understandable that disease control workers’ turnover rate is relatively high. The turnover rate among these control workers in South Korea is as much as five times higher than the average rate of Korean public officers (i.e., 6.9% and 1.4%, respectively [4]).

Furthermore, the likelihood that Korean FMD/AI control workers’ will seek the professional help of their own free will is quite slim, not to mention there are few professional services to choose from nationwide [1]. However, this poor help-seeking behavior among these Korean workers is similar to behaviors observed worldwide. For example, in previous research on FMD workers in the United Kingdom, relatively few people approached health or social services, mainly because they did not view their reaction as a problem or illness [5]. However, in Korea, the Nation Human Rights Commission of Korea [6] announces that there being no follow-ups at the organizational or national level after the event is a violation of human rights for not taking care of possible mental health impacts.

There are currently few specialized methods to help with their occupational distress. The present study aims to gain a deeper understanding of the mental health statuses of FMD/AI workers who participated in an EAL program and to test for changes in their stress level, coping skills, and overall health. The rationale behind using Equine Assisted Learning (EAL) for this population in this study is that equine-assisted activities are suggested to improve participants’ various levels of physical, emotional, psychological, and social needs [7,8,9,10]. We applied EAL to Korean FDM/AI control workers. We explored its usefulness by examining participants’ improvement in overall functioning, including reduced stress and enhanced coping styles, as well as improvements in various quality of life domains. Hence, it is exploratory research.

### 1.1. Mental Health Issues of Korean FMD/AI Control Workers

Epidemic control workers belong in the same group as any other disaster workers, including firefighters, police officers, medical personnel and paramedics, search and rescue workers, transport and recovery personnel, mental health and social services personnel, and volunteers who assist with high-risk situations [11]. The nature of their trauma and frequent exposure to it is directly related to their job, resulting in a significant impact. The lingering effect of such traumatic scenes may also overlap in other situations where some disaster workers have difficulty detaching their traumatic issues and confuse the living animal with their family/loved ones and vice versa. In addition, disaster workers experience secondary trauma associated with this task, including anxiety, irritation, disinterest, helplessness, indifference, sweating, negative disposition, substance use, compassion fatigue, and vicarious victimization [11,12]. Thus, one issue for disaster workers is that the job involves frequent exposure to traumatic scenes that can repeatedly evoke negative emotions.

Recently, the National Human Rights Commission of Korea appointed the Seoul National University Institute for Social Development to examine the psychological health status of 268 public officials and veterinarians who participated in the killing of animals while performing FMD control. Using an 11-point scale of severity of difficulty/discomfort, the mean score was 9.02, indicating that the labor intensity of the work was relatively high. In addition, 76% of participants exhibited a clinically diagnosable level of PTSD. These findings are assumed to be associated with 43 accidents that transpired between 2000 and 2017 when FMD was aggressively taking place [6]. A similar report suggested high levels of PTSD symptoms among these workers (i.e., a mean score of 41 points, with a diagnostic cut-off score of 24 points) and more significant negative emotions compared to average Koreans [13]. Given that the impact of trauma is relatively greater among persons who have observed a disaster directly or have been physically harmed compared to those who have not, it is understandable that frontline workers scored high [14].

Fundamentally, FMD/AI control work is far more severe than ordinary office “work” because the job description is shocking [2]. Particularly when deliberately killing living creatures, there is a high probability of experiencing internal conflict over personal and moral values, irrespective of personal choice [4]. This experience can induce extreme stress, negative emotions, nightmares or hallucinations, and in severe cases, PTSD symptoms, not to mention the nature of traumatic scenes experienced by all sensations (e.g., seeing, hearing, smelling). Therefore, FMD/AI control workers are far more prone to experiencing fear, helplessness, negative affect, and avoidance. Although these negative responses are considered quite natural and can even be predicted before participating in the task [12], they must be resolved with extreme care and at a professional level. Given the inability to entirely prevent and/or control natural disasters every year in South Korea, it is important to establish a psychological support system and have an effective employee program that can promptly and efficiently respond after every traumatic event.

### 1.2. Importance of Stress Coping

When faced with difficult or stressful situations, how people process stressors is critical in determining whether trauma—or the level of stress resulting from critical events—will be experienced. According to various stress hypotheses, it is relatively easy to overcome if an individual can deal with stressful situations through good coping skills and a healthy support system [15,16]. For example, although all veterans experience war, not every veteran presents symptoms of PTSD. The problem, however, is that anxiety or depression can be expected and temporary reactions, but if stressors are too excessive (e.g., disasters, trauma, death of a loved one), repetitive, or persist for an extended period, the individual’s ability to cope with the stressful situation is significantly weakened [17].

Stress coping can be learned in overcoming challenging situations to regain a more stable emotional state, hence a critical behavioral approach in counseling and therapy. In neuroscience, this process is associated with serotonin function, where the difficulties of human emotions are regulated through hormonal balance. Interestingly, serotonin release can be manipulated by conditioning our brain into peaceful or healing situations, such as listening to soothing music or thinking about enjoyable images or landscapes, known as “greenness effects” [18]. In addition, behavioral activities, such as walking, hiking, and exposure to daylight, are some practical non-pharmacological methods for maintaining one’s serotonin system at a stable level [19].

### 1.3. Usefulness of EAL for Individuals Experiencing Stress and Trauma

EAL is a form of Equine Assisted Activities and Therapy (EAAT) that aims to help individuals cope with personal emotional and behavioral problems through a learning-based program [8]. Although resonating with some of the core values found in other equine-guided interventions (e.g., equine-assisted psychotherapy, therapeutic riding), EAL is an educational program that is facilitated using a group format and provides opportunities for individual attention during each session [20]. In EAL, participants engage in structured, facilitator-led sessions with constant feedback related to participants’ experiences. In addition, group sessions provide opportunities for participants to become involved in situations that require interaction, not only with the horse but also with group members, and the opportunity to reflect on these experiences. Therefore, EAL provides experiential learning to increase participants’ social functioning through unmounted (ground) and mounted (riding) activities to gain insight, self-awareness, empathy, confidence, a sense of accomplishment through problem-solving, and increased interpersonal skills [20]. Hence, EAL is different from casual horseback riding or simple rapport training.

The trained horse is generally considered the teacher, and a human facilitator helps guide the participants during the process of learning and understanding. Horses have a significant advantage over other animals in that they are more sensitive to the emotional state of humans and the movement of their external environment than any other animals used for mediated therapy [21]. Horses mirror the rider’s emotional state [21]; for example, if the rider is sitting on his back and is nervous, the horse being ridden is also tense). In a riding context, horses tend to depend on their leader (i.e., the rider) and tune in to verbal and non-verbal cues given by the rider. Because horses are known to be the only animals that can provide immediate feedback on the emotional state of humans [21], whether the horse is ridden or unridden, rehabilitation is expected to have significant psychological and emotional benefits through interactive communication with horses. Therefore, equines have been included in a range of therapeutic human service contexts, including counseling and learning programs in correctional facilities [22,23], mental health facilities [24], social services [25], women’s and youth addiction treatments [26], and high-risk youth and veterans who have experienced emotional and behavioral trauma [27,28,29,30].

Research shows that EAL is effective for reducing trauma symptoms [29,31], promoting hope and reducing depression in at-risk youths [9], increasing attachment, confidence, empathy, communication, and social skills in adolescents, as well as increasing self-control, greater family intimacy, and lower impulsivity in juveniles with attention deficit hyperactivity disorder (ADHD) traits [32]. Most recently, in South Korea, participation in EAL was associated with reduced stress levels in probation officers and PTSD symptoms among firefighters in South Korea [33], who are similar in the type of work that involves high stress to FMD/AI control workers.

## 2. Methods

### 2.1. Study Design and Participants

This study incorporated a non-randomized pre-and post-test design using a single treatment group of 51 voluntary study participants. Recruitment flyers were sent out to public officials (whose job specification includes FMD/AI control work) with the help of the Korean Ministry of Agriculture, Food, and Rural Affairs. Prospective participants then registered with a nearby contracted Therapeutic Riding Center. These centers run the same EAL program curriculum so that participants are provided the same program throughout Korea. We originally anticipated at least 40 voluntary participants with a maximum of 80 subjects. Before the first session, the study purpose and methods were thoroughly explained, and a written consent form was provided, outlining a free-of-charge program, voluntary participation, anonymity, and collection and use of personal information. Moreover, the current study was approved by the Institutional Review Board of Jeonbuk Horse Industrial Complex Center. The questionnaire for our pre and post-test was conducted via an encrypted online survey link sent individually to participants and required a password and smartphone. A printed version of the questionnaire was distributed, sealed, and passed to the primary investigator for coding and analysis in a few cases where the individual could not use a smartphone. The collected data file was encrypted, stored under lock and key, and was shared only with the researchers responsible for the coding, cleaning, analyzing, and writing documents. The entire program was free of charge, and no physical or psychological discomfort occurred during data collection. Any person with a current mental health diagnosis was excluded from the study.

### 2.2. EAL Program

The EAL program we implemented in this study was specifically developed by a multidisciplinary research team affiliated with the Therapeutic and Healing-Riding Center at Korea Racing Authority (KRA) and with the advice of PATH Intl’s licensed international experts. The same program was previously applied to and evaluated the effectiveness and usefulness among Korean probation officers and firefighters. The use of EAL program on Korean firefighters has been supported by a recent study [33] and other studies on the applications of EAL programs on military veterans [29]. Applying EAL to FMD/AI control workers may be somewhat controversial and needs careful implementation, given the high level of stress and negative emotions evoked in the nature of work in animal-involved disaster settings. However, if the program is used correctly by trained and licensed professionals, as in this study, this equine intervention can help reduce stress and improve the coping skills of these individuals in high-stress work, unlike desensitization processes in trauma therapies.

The EAL program used in this study consisted of eight weeks of a total of 16 sessions, six sessions of unmounted (ground) activities, and ten sessions of mounted (riding) activities. Ground activities included observing horse behaviors, grooming, leading, desensitizing, and harnessing. Instructors were all trained and licensed professionals (by PATH International and Korean Therapeutic Riding Licensure) with at least 5 years of experience in therapeutic riding. EAL programs were conducted as group sessions with 2–3 participants per instructor. Although assigned horses may change during the course of the program, the same instructor remained from start to finish of the program.

The main goal of the riding activities was to help participants acquire independent riding skills by getting on the back of a horse and gradually learning to walk and trot. In addition, these activities aimed to increase self-control, accomplishment, and problem-solving ability. See Table 1 for specific program schedules and components.

### 2.3. Materials and Methods

Health and mental health domains related to characteristics of the high-risk stress jobs of FMD/AI control workers were our primary interest, including the perceived stress level, coping strategies, and overall health statuses. Demographic questions included age, gender, marital status, highest education level attained, religion, length of employment in current service, hobby, participation in one or more livestock killings, and severity of emotional pain.

### 2.4. Perceived Stress Level: Korean Version of the Perceived Stress Scale (KPSS)

We used the KPSS to examine our participants’ perceived stress levels and observe changes before and after participating in the program. The KPSS, standardized by [34] and previously developed by [35], consists of 10 items rated on a 5-point Likert scale, with responses ranging from 0 (never) to 4 (very often). Scores are summed across all scale items, with higher scores indicating higher levels of perceived stress. Total scores of 12 or below are considered normal, scores 13–15 are considered mild, scores 16–18 are considered moderate, and scores 19 and above indicate high Stress [36]. Cohen [35] reported good internal reliability (α = 0.78) for the original scale, and Hur and Rlee [37] reported α = 0.72. Cronbach’s α in the present study was 0.81 at the pre-test and 0.85 at the post-test.

### 2.5. Coping: Korean Version of the Coping Strategy Indicator (K-CSI)

We used the K-SCI, originally developed by Amirkhan [38] and translated by Shin and Kim [39], to assess three basic orthogonal coping modes: problem-solving, seeking social support, and avoidance. Higher scores on a strategy indicate greater use of that strategy. The CSI consists of 33 items rated on a 3-point scale (1 = not at all; 2 = a little; 3 = a lot). CSI has been used to assess coping strategies for various populations in various contexts [40,41,42,43,44]. Shin [45] reported fairly good internal reliability for all three subcategories: seeking social support α = 0.80; problem-solving α = 0.87; and avoidance α = 0.68. For the present study, Cronbach’s α was: α = 0.87 and 0.89 for seeking social support; α = 0.89 and 0.90 for problem-solving; and α = 0.78 and 0.80 for avoidance, at pre and post-test, respectively.

### 2.6. Quality of Life (Mental Health and Vitality): SF-36 Survey

The SF-36 health survey [46] has been widely used to assess the quality of life in eight health domains, including limitations on (1) physical activities due to health problems, (2) social activities due to physical or emotional problems, (3) usual role activities due to physical health problems, (4) bodily pain, (5) general mental health (psychological distress and well-being), (6) limitations in usual role activities due to emotional problems, (7) vitality (energy and fatigue), and (8) general health perceptions. Higher scores indicate a better perception of functional health and well-being. Cronbach’s α in the original study ranged from 0.64 to 0.96 [46]. The scale was translated and validated into Korean by Nam [47] and showed good overall reliability (α = 0.89) [48]. Cronbach’s α in the present study was α = 0.89 and 0.90 for physical activities due to health problems; α = 0.56 and 0.68 for social activities due to physical or emotional problems; α = 0.63 and 0.78 for usual role activities due to physical health problems; α = 0.60 and 0.69 for bodily pain; α = 0.84 and 0.85 for general mental health (psychological distress and well-being); α = 0.71 and 0.75 for limitations in usual role activities due to emotional problems; α = 0.73 and 0.82 for vitality (energy and fatigue); and α = 0.75 and 0.79 for general health perceptions at pre-and post-test, respectively.

### 2.7. Statistical Analysis

In addition to a descriptive analysis of demographic information of 45 program participants (6 cases were deleted due to unfaithful and missing values), a paired t-test was performed to observe any changes in the main variables of interest after the EAL program was completed. An analysis of covariance (ANCOVA) was also conducted to determine whether there were differences in quality of life (mental health and vitality)—after the EAL program was completed—across age, length of service, and massive culling experience. All quantitative analyses were performed using Predictive Analytics Software (PASW) 18.0. The significance level for the study was set to *p* < 0.05.

## 3. Results

### 3.1. Description of Study Participants

The mean age of the participants was 41.2 years (SD = 10.5), with the majority of program participants being male (84.4%) and having attended university or a higher level of education (82.2%). The mean length of employment in the current service was approximately 119.1 months (SD = 124.4), or ten years. Half of the participants (53.3%, n = 24) had less than five years of employment, and about 22.2% (n = 10) were employed at the same workplace for more than 20 years.

Of our 45 participants, 75.6% (n = 34) of respondents had one or more livestock killing experiences. When responding about the difficulty of the killing incident, almost everyone (97.1%, n = 33) recalled the massive culling experiences as difficult, with 1 out of 3 reporting (38.2%, n = 13) that it was ‘very difficult.’ When asked about any psychological help they had received in the past to cope with the emotional pain associated with their trauma, only a few participants (8.8%, n = 3) reported receiving any treatment. However, this treatment mostly consisted of medical attention related to physical injury (e.g., arm ligament or back pain) and was not associated with psychological trauma. Of the 34 respondents with one or more such experiences, only one had received professional counseling and had taken prescribed medication previously for anxiety and lack of self-control for the past three years. These results indicate that very few of these control workers sought professional help, despite their self-report of experiencing emotional difficulty from reminiscing about the killing incidents.

### 3.2. Changes in Quality of Life

**Figure 1:**
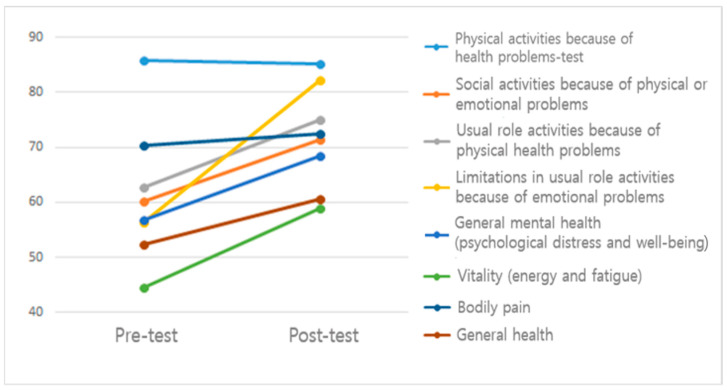
Quality of life before and after EAL program participation (N = 45).

Table 2 and Figure 1 show that participants’ overall quality of life improved in six health domains measured by the SF-36 health survey. Most improvement was observed in the limitations in usual role activities due to emotional problems (t = −5.23, *p* < 0.01, 46.0% improvement after EAL program participation) and vitality (energy and fatigue) (t = −6.71, *p* < 0.01, 32.1% improvement), followed by general mental health (psychological distress and well-being) (t = −5.57, *p* < 0.01, 20.4% improvement), social activities due to physical or emotional problems (t = −4.07, *p* < 0.01, 18.4% improvement), usual role activities due to physical health problems (t = −3.17, *p* < 0.01, 19.4% improvement), and general health (t = −3.21, *p* < 0.01, 15.6% improvement). Based on effect sizes, the vitality domain had the greatest level of improvement (Cohen’s d = 1.00), followed by general mental health (0.83), limitations in usual role activities due to emotional problems (0.78), social activities due to physical or emotional problems (0.61), general health (0.45), and usual role activities due to physical health problems (0.47).

The ANCOVA showed a difference in the post-test mean on physical activities due to health problems and vitality after controlling whether participants had one or more massive culling experiences in the past. More specifically, individuals with one or more enormous culling experiences (Adjusted Mean = 71.5, SE = 3.4) showed lower levels of physical activities due to health problems compared to their counterparts with no tremendous culling experience (Adjusted Mean = 86.0, SE = 6.0). Likewise, individuals with one or more massive culling experiences (Adjusted Mean = 56.3, SE = 2.2) showed lower vitality levels than their counterparts with no tremendous culling experience (Adjusted M = 67.0, SE = 3.9). In addition, the ANCOVA results indicated that participants with five or more years of employment in current service (Adjusted Mean = 55.6, SE = 3.2) showed lower general health levels at post-test than those under five years of a career (Adjusted Mean = 64.9, SE = 3.0).

### 3.3. Changes in Perceived Stress Level

Table 3 shows a significant change in perceived stress level after program participation (t = 4.77, *p* < 0.001). When assessing across stress level categories, there was a 17.8% decrease in participants requiring clinical assessment for their depression or anxiety after program participation (from 68.9%, n = 31, to 51.1%, n = 23). Although only four participants (8.9%) were in the normal stress range (i.e., a score of 13 or lower) before program participation, there was a 15.5% increase from 4 to 11 participants (24.4%) in the normal stress range after participation. Among the 22 participants (48.9%) who exhibited high-stress levels before program participation, 13 (59.1%) showed improvement.

The ANCOVA revealed a significant difference in the post-test mean scores on stress level after controlling for whether participants had one or more massive culling experiences in the past. More specifically, individuals with one or more massive culling experiences (Adjusted Mean = 15.2, SE = 0.60) exhibited a higher stress level than their counterparts with no massive culling experience (Adjusted Mean = 13.8, SE = 1.1).

### 3.4. Changes in Stress Coping

See Table 4 for changes in the three domains of stress coping strategies (i.e., problem-solving, avoidance, and social support seeking). Participants’ problem-solving skills slightly increased (from M = 4.10, SD = 0.61 to M = 4.20, SD = 0.63), and their avoidance tendency significantly decreased (from M = 4.15, SD = 0.62 to M = 3.80, SD = 0.57). In addition, social support seeking behaviors also improved (from M = 4.06, SD = 0.61 to M = 4.39, SD = 0.65). However, this improvement was not statistically significant. In terms of effect sizes, Cohen’s d for social support seeking was 0.57 and for avoidance was 0.56, indicating moderate effect sizes for the program across the two coping domains. However, no differences were observed across previous massive culling experiences or length of employment in current service.

## 4. Discussion

In summary, (1) the overall stress levels of our participants were significantly reduced after participating in the EAL program; (2) participants’ tendency to pursue problem-solving based strategies and social support improved while using less avoidance-oriented strategies; and (3) positive changes were observed in various life domains, including both mental health and physical aspects (e.g., vitality). Differences in quality of life, depending on whether participants had one or more massive culling experiences in the past, especially in terms of stress level, physical activities due to health problems, and vitality, were also essential findings. For example, although the mean stress level score decreased after program participation, individuals with one or more massive culling experiences remained at a higher stress level than those without such experiences. This finding may indicate the unresolved nature of such traumatic experiences, and these workers likely require additional professional help to cope with their psychological and/or emotional issues.

The observed changes in coping styles were our most important finding. Participant avoidance tendencies diminished, while problem-solving and help-seeking behaviors improved. We may explore how this occurred by examining the program components and the uniqueness and strengths of equine-assisted activities, especially building resilience. Resilient people tend to deal with stress without falling apart or running away (as in fight-or-flight response) and seek healthy solutions for overcoming stressful situations. High resilience can be learned in overcoming frustration, whereby individuals encounter various experiences that allow them to regain confidence and maintain a stable emotional state.

Frederick, Hatz, and Lanning [9] showed even a brief (5-week) intervention of non-riding EAL positively impacted the lives and attitudes of at-risk youth. Veterans with PTSD reported learning new and/or enhanced communication and conflict resolution skills that gave them hope for improved personal relationships as they move forward and reduced PTSD symptoms during the EAL session, suggesting that the horse activities elicited positive experiences [29]. It is important to note that we also observed differences in vitality level depending on whether participants had massive culling experiences in their past. Even though the overall quality of life improved after program participation, vitality was still lower among those with one or more traumatic experiences than those without such experiences. This type of psychological lethargy can result in lower self-efficacy; individuals have lower expectations and do not believe they can do something successfully. Indeed, victims of domestic violence who feel they cannot escape their violent situation are just examples of learned helplessness. From this point of view, participants who experienced massive culling may be unable to recover from these shocking events because they have not received psychological and emotional support for their trauma. Thus, their quality of life may still be lower than others, even after completing the EAL program, due to their negative antecedent experiences.

Although using EAL may be considered controversial, given that the high stress of FMD/AI control workers involves killing animals, our findings suggest that if used correctly by trained professionals, this equine intervention helps reduce stress and improve the coping skills of these individuals. Although our participants are not representative of all psychological states experienced by similar workers, as our sample size was too small to represent the whole population, we did find improvement in the lives of one of the most high-risk occupational stress groups.

## 5. Conclusions

Epidemic control work is a professional effort to prevent and decrease the risk of developing or spreading disease. However, this task is inevitably performed by humans who must brutally kill livestock, resulting in significant negative physical, psychological, mental, and social impacts on individuals and communities involved in the process. These negative impacts include extreme stress, anxiety, and moral confusion, to name a few; therefore, it is a “disaster” for everyone.

In the present study, we explored the usefulness of an EAL program to improve various life domains of FMD/AI control workers in South Korea. According to our findings, EAL effectively improved stress and increased healthy coping styles in program participants. In particular, positive changes for coping with stress were a significant outcome, as it is related to resilience, which is the ability to cope with future stressors healthily. 

Despite statistically significant findings of the study, caution needs to be taken in making definite conclusions about the impact of the occupational environment that lead to high stress levels and the effectiveness of the EAL program on improving stress levels and quality of life. Moreover, given that this study was conducted in more of a pilot form and did not follow up on the subjects after the program, there is little evidence to suggest that their increased coping skills had a long-term effect. However, given the results, we have gained some understanding of horses’ use in helping humans become mentally healthy in stressful situations. Equine-assisted approaches such as the EAL program used in our study may be adopted by different countries regardless of differences in culture or language because horses have their unique ways of non-verbally yet efficiently communicating and coordinating with human behaviors. Future studies should include greater sample sizes, conduct follow-up studies, and explore other mental health needs, such as alcohol or drug use, as these are two common comorbidities alongside PTSD, anxiety, and depression. Comparison with a control group would be necessary to rule out any external factors that may explain the effectiveness of the EAL program. In terms of future research from a strength perspective, it may be valuable to investigate protective factors (e.g., resiliency, social support) and determine how EAL reinforces protective factors to improve well-being. 

## Figures and Tables

**Table 1:** Description of EAL program.

	Session	Themes	Horse Skills	Life Skills
Ground Activities	1	Orientation	Program introduction schedule, riding etiquette, safety training (emergency response)	New acquaintance relationship
	2	Horse Behaviour Observation	Understanding and communicating with horses (non-verbal, action) Putting on a headcollar	Social ability acceptance
	3	Grooming	Reasons for grooming, understanding grooming kit, observation/understanding horse body structure	Remembering order hygiene
	4	Leading	Introduction to riding aids, using a headcollar and leading with a lead rope	Leadership/fellowship accomplishment
	5	Desensitization	Understanding desensitization, horse traits and behaviors, desensitivity activities	Building trust and communication skills
	6	Harnessing	Introduction to harnessing tools, how and when precautions and management	Remembering order skills
Mount Activities	7	Getting on/off the horse	Riding and getting off the horse, posture/position/stirrups, precautions and emergency response	Confidence accomplishment, problem-solving
	8	Walk and stop	Basic riding posture, practice walk and stop, practice riding aids	Self-control responsibility
	9	Walk with control	Change of direction, halt, course activity, group riding etiquette, speed (gait) control	Control acquiring etiquette
	10	Walk and vaulting exercise	Warm-up before vaulting, walk rhythm, repetition of 8–9th session	Concentration Confidence
	11	Walk and trot 1	Understanding trot rhythm, using correct trot aids,	Challenging physical development,
	12	Walk and trot 2	speed (gait) control, practice trot and stop, correct posture, repetition	accepting and alternating independence
	13	Making turns while trotting	Introducing circles, bending, lunging	Partnership physical development
	14	Walk and trot course	Course work	Problem-solving Communication skills
	15	Walk and trot game	Course work	Creativity Motivation
	16	Last session, independent riding	Review of previous learning, independent riding and feedback	Goof effort Emotion control

**Table 2:** Quality of life before and after EAL program participation (N = 45).

Subcategories	Before	After	T	P	Cohen’s D
	M(SD)	M(SD)			
Physical activities because of health problems	85.9 (17.5)	85.1 (16.2)	0.405	0.69	0.06
Social activities because of physical or emotional problems	60.3 (17.9)	71.4 (13.5)	−4.07	0.001	0.61
Usual role activities because of physical health problems	62.8 (34.4)	75.0 (27.2)	−3.17	0.003	0.47
Limitations in usual role activities because of emotional problems	56.3 (39.5)	82.2 (31.5)	−5.23	0.001	0.78
General mental health (psychological distress and well-being)	56.9 (13.7)	68.5 (12.4)	−5.57	0.001	0.83
Vitality (energy and fatigue)	44.6 (14.3)	58.9 (16.2)	−6.71	0.001	1.00
Bodily pain	70.3 (17.0)	72.4 (15.9)	−0.93	0.36	0.14
General health	52.4 (16.3)	60.6 (16.9)	−3.21	0.002	0.48

**Table 3:** Changes in perceived stress level (N = 45).

	Pre	Post	T	P	Cohen’s D
	M(SD)	M(SD)			
Perceived stress level	17.8 (4.4)	14.9 (4.2)	4.77	<0.001	0.24

**Table 4:** Changes in Stress coping (N = 45).

Subcategories	Pre	Post	T	P	Cohen’s D
	M(SD)	M(SD)			
Social support seeking	4.06 (0.61)	4.39 (0.65)	−2.002	0.052	0.57
Problem-solving	4.10 (0.61)	4.20 (0.63)	−3.28	0.002	0.16
Avoidant	4.15 (0.62)	3.80 (0.57)	2.72	0.023	0.56

## Data Availability

Not applicable.
